# Rechallenge of denosumab in advanced giant cell tumor of the bone after atypical femur fracture: A case report and review of literature

**DOI:** 10.3389/fonc.2022.953149

**Published:** 2022-07-19

**Authors:** Vincenzo Nasca, Anna Maria Frezza, Carlo Morosi, Ciriaco Buonomenna, Antonina Parafioriti, Giorgio Zappalà, Federica Bini, Paolo Giovanni Casali, Mattia Loppini, Silvia Stacchiotti

**Affiliations:** ^1^ Department of Medical Oncology, Fondazione Instituti Ricovero e Cura a Carattere Scientifico (IRCCS) Istituto Nazionale dei Tumori, Milano, Italy; ^2^ Department of Radiology, Fondazione Instituti Ricovero e Cura a Carattere Scientifico (IRCCS) Istituto Nazionale dei Tumori, Milano, Italy; ^3^ Department of Pathology, Aziende Socio Sanitarie Territoriali (ASST) Pini – Centri Traumatologici Ortopedici (CTO), Milano, Italy; ^4^ Department of Orthopaedic Surgery, Ospedale Papa Giovanni XXIII, Bergamo, Italy; ^5^ Department of Medical Oncology, Ospedale Papa Giovanni XXIII, Bergamo, Italy; ^6^ Department of Oncology and Hemato-oncology, University of Milan, Milano, Italy; ^7^ Department of Biomedical Sciences, Humanitas University, Milano, Italy; ^8^ Department of Orthopaedic Surgery, Instituti Ricovero e Cura a Carattere Scientifico (IRCCS) Humanitas Research Hospital, Milano, Italy

**Keywords:** denosumab, giant cell tumor of bone, sarcoma, atypical femur fracture, bone tumor

## Abstract

Giant cell tumor of the bone (GCTB) is a locally aggressive neoplasm where surgery is often curative. However, it can rarely give rise to distant metastases. Currently, the only available active therapeutic option for unresectable GCTB is denosumab, an anti-RANKL monoclonal antibody that dampens the aggressive osteolysis typically seen in this disease. For advanced/metastatic GCTB, denosumab should be continued lifelong, and although it is usually well tolerated, important questions may arise about the long-term safety of this drug. In fact, uncommon but severe toxicities can occur and eventually lead to denosumab discontinuation, such as atypical fracture of the femur (AFF). The optimal management of treatment-related AFF is a matter of debate, and to date, it is unknown whether reintroduction of denosumab at disease progression is a clinically feasible option, as no reports have been provided so far. Hereinafter, we present a case of a patient with metastatic GCTB who suffered from AFF after several years of denosumab; we describe the clinical features, orthopedic treatment, and oncological outcomes, finally providing the first evidence that denosumab rechallenge after AFF occurrence may be a safe and viable option at GCTB progression.

## Introduction

Giant cell tumor of the bone (GTCB) is a rare mesenchymal neoplasm, accounting for <5% of tumors directly arising from the bone. It typically affects the long bones, but may also arise from the axial skeleton, in particular from the sacrum or vertebrae (1). GTCB is regarded as a “rarely metastasizing” entity, according to the WHO classification, usually presenting with locally aggressive features, behaving as a mass progressively enlarging and destroying the bone and invading surrounding structures (2, 3). Distant metastases, mainly pulmonary, are reported in <5% of all GCTB cases. Histologically, GCTB consists of three different cellular populations: receptor activator of nuclear factor-kappa beta ligand (RANKL)-expressing ovoid stromal cells, which are the true neoplastic cells that actively recruit the other two populations into the tumor microenvironment, and osteoclastic multinucleated giant cells expressing RANKL and RANK-expressing myeloid mononuclear cells. The osteoclast-like giant cells are responsible for the typical aggressive osteolysis seen in this disease. This is accomplished *via* the RANK–RANKL axis, a process that physiologically serves in bone remodeling and osteoclastic differentiation and activity, and which is upregulated in GCTB, ultimately inducing bone resorption (4–8).

For a localized, resectable disease, surgery is the standard treatment, with different possible approaches (intralesional curettage with or without adjuvant therapy and en bloc excision) depending on tumor primary site, size, and involvement of surrounding soft tissues, which is the most relevant risk factor for local recurrence (1, 2). Of note, compared to local curettage, en bloc resection yields a lower recurrence rate, which nonetheless remains significant (up to 15%) (9–13).

For many years, patients with locally advanced, unresectable, or metastatic GCTB had limited, unsatisfactory treatment options, until the availability of denosumab, a fully humanized monoclonal antibody (mAb) that binds to and inhibits RANKL, therefore blocking the RANK–RANKL axis, halting the formation of osteoclastic giant cells, and finally dampening the osteolytic process (14–16). The introduction of denosumab revolutionized the therapeutic landscape of GCTB. Denosumab’s safety and efficacy in this setting were demonstrated in an international phase II trial and confirmed in the real-world setting, obtaining durable, objective responses in the vast majority of patients (17–19). It currently represents the standard treatment for metastatic or technically unresectable disease, with a well-known, generally acceptable short-term toxicity profile, with grade 3 adverse events occurring in approximately 20% patients and rarely reported serious adverse events. Much less is known about denosumab’s long-term toxicity profile and safety, which is relevant in this setting where denosumab treatment might be required lifelong (13, 16, 20). Uncommon but serious complications from denosumab treatment are osteonecrosis of the jaw (ONJ) and atypical femur fracture (AFF). AFF’s incidence reported in trials is low (approximately 1%), but, as it usually occurs after multiple years of treatment and data with patients on long-lasting therapy is limited, its true incidence may be underestimated (17, 18).

When AFF occurs, current guidelines recommend prompt discontinuation of denosumab and appropriate orthopedic treatment (21, 22). Currently, no data are available on the feasibility of denosumab rechallenge after AFF resolution in patients with GCTB progression.

We herein reported the case of a patient suffering from a metastatic GCTB and developing AFF after several years of denosumab treatment. We described the clinical presentation, the orthopedic approach, and oncological outcomes, showing how denosumab could be safely restarted at the time of disease progression.

## Case presentation

**Figure 1:**
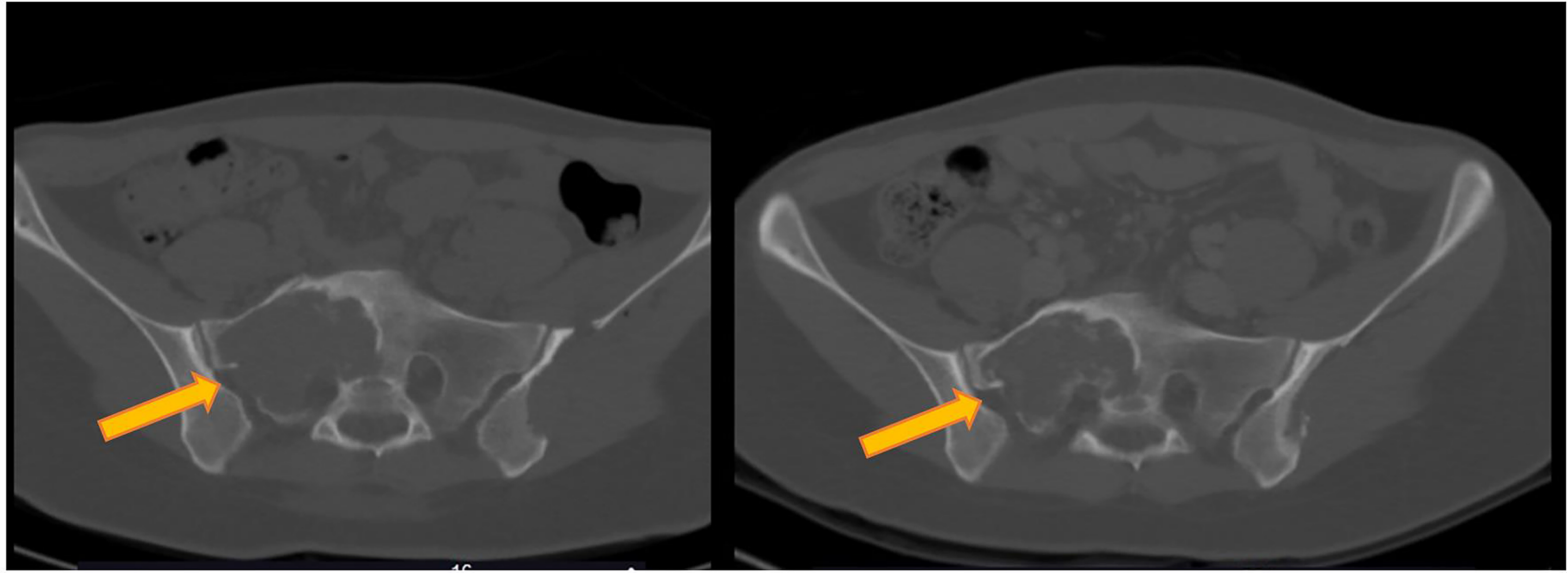
Response of target lesion (located in the sacrum) to denosumab in a metastatic giant cell tumor of the bone (GCTB). Left panel’s image is a CT scan showing a sacral lesion at baseline, and, on the right panel, a CT scan after 3 months of denosumab treatment showing a reduction in tumor size (partial response according to RECIST 1.1).

**Figure 2:**
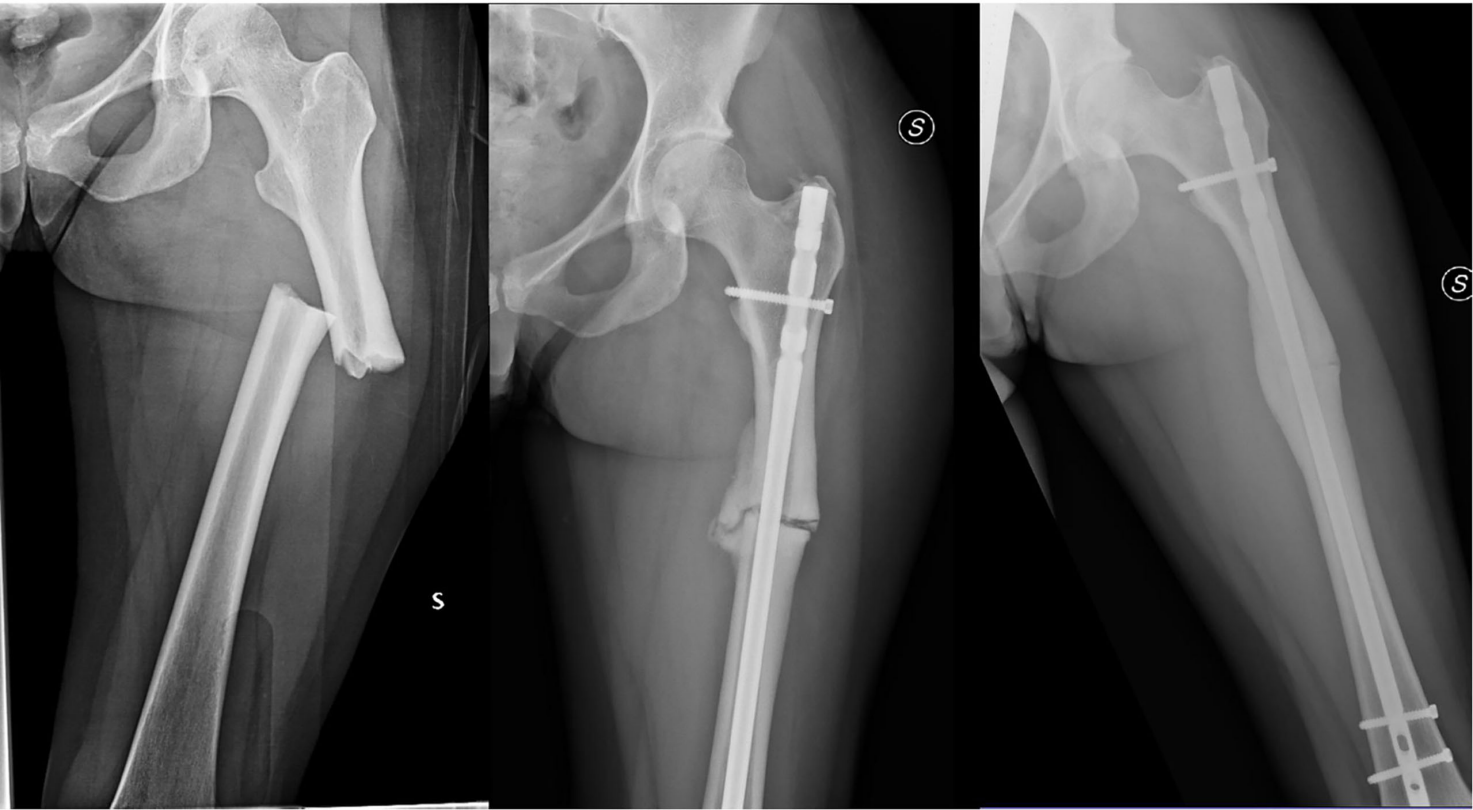
Plain X-ray showing complete, displaced femoral diaphyseal fracture (left panel), reported as an atypical femur fracture (AFF). Patient was treated with open reduction and intramedullary nailing, but bone healing was delayed (plain X-ray after 5 months from surgery, central image). Bony callus eventually fully consolidated after 13 months (right panel).

**Figure 3:**
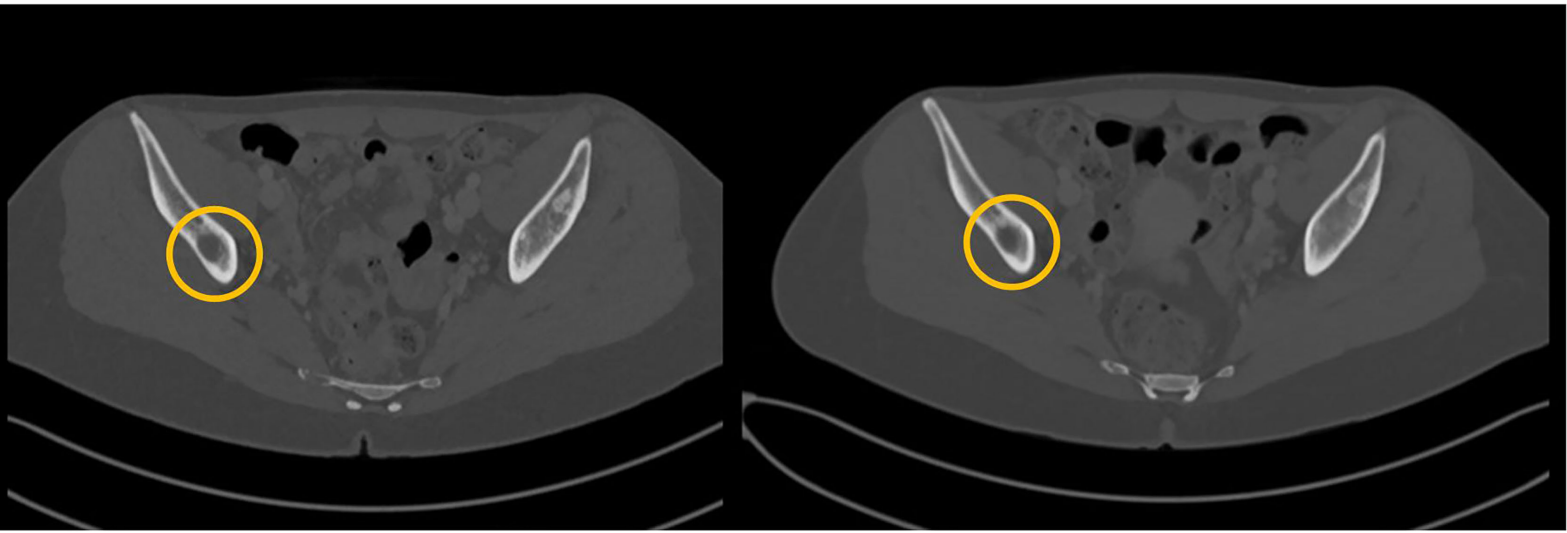
After 14 months from denosumab discontinuation due to AFF, progressive disease was documented (new lesions in right iliac wing and L5 soma, left panel) and denosumab restarted. On the right panel, a CT scan obtained 3 months later shows stabilization of disease (stable disease according to RECIST 1.1).

An otherwise healthy 20-year-old woman was diagnosed with GCTB in February 2009, after an accidental fall while skiing. X-Ray examination showed an osteolytic area in the proximal tibia. The lesion was resected *via* curettage, and histological examination was positive for GCTB. Subsequent systemic staging highlighted a 5-cm large sacral primary (**Figure 1**, left panel), multiple bone (D9-11, L3, L5, right hemi-sacrum, and right femur) metastases and one single pulmonary metastasis in the superior lobe of the left lung. Diagnosis of GCTB was histologically confirmed on the sacral lesion. Family history was negative for cancer, and patient had no comorbidities at the time of the event. The patient was symptomatic for diffuse back and buttock pain, invalidating her normal daily activities and gradually determining an impairment in walking, which finally constrained her to the use of a wheelchair. Given the diagnosis and the disease extent, in November 2009, she was started with denosumab (120 mg subcutaneously, once per month, following the loading dose of 120 mg day 1-8-15), obtaining a reduction in tumor size and resolution of all GCTB-related symptoms (**Figure 1**, right panel). The disease response and clinical benefit were maintained for over 10 years. After 132 months of treatment (November 2019, at the age of 30), the drug was interrupted due to a fracture of the left femur diaphysis, after a minor trauma (low-energy fall while walking). The fracture carried the typical clinical and radiological features of a complete displaced AFF (**Figure 2**, left panel), which was related to denosumab. The patient was treated with open reduction and intramedullary nailing, along with calcium and vitamin D daily supplements, to accelerate fracture healing (**Figure 2**, right panel). However, bony callus fully consolidated only 13 months after the nailing. GCTB remained stable for 14 months after denosumab discontinuation, when a new CT scan showed progressive disease by RECIST, marked by the appearance of two new lesions in the right iliac wing and in L5 soma (**Figure 3**, left panel). After multidisciplinary evaluation and orthopedic assessment, denosumab was restarted at the previous dosage (120 mg per month, subcutaneously). At the first re-assessment, 3 months later, a new tumor stabilization was documented and confirmed at 1 year, with no evidence of AFF relapse or additional serious adverse events reported (**Figure 3**, right panel).

## Discussion and literature review

We reported on a patient presenting with a highly symptomatic, metastatic GCTB, treated with denosumab, and achieving a disease response and clinical benefit maintained for several years, who eventually developed a denosumab-related AFF, a rare but serious adverse event associated with long-term treatment with denosumab. Denosumab was interrupted, and the fracture could be surgically managed and cured, although with a significantly prolonged healing time. As disease progression occurred after 14 months from denosumab discontinuation, the treatment was restarted, achieving a new response and with no further treatment-related toxicity after 1 year. To our knowledge, this is the first report suggesting that denosumab rechallenge after AFF resolution is a feasible option in patients with advanced, progressive GCTB.

Treatment of advanced GCTB relies on denosumab, which drastically improved prognosis and quality of life in these patients (17, 18). The safety and efficacy of this drug were first reported by a multicenter phase 2 study (NCT00680992) and subsequently confirmed (17, 18). These studies showed long-lasting disease control and prolonged clinical benefit in unresectable patients. However, the long-term major side effects and how to handle major toxicity are not well described. On the other side, there is no consensus about denosumab discontinuation in advanced/metastatic GCTB. There is evidence showing that at least nearly one-half of patients do experience disease progression when the drug is discontinued, after a median of <1 year (17, 20). On the other hand, there are only anecdotal reports of effective denosumab rechallenge at GCTB recurrence/progression so that more data are needed (13). As long-term treatment with denosumab may be associated with serious adverse events, like osteonecrosis of the jaw (ONJ) and AFF, they need to be promptly recognized by physicians (17). In addition, to our knowledge, there are no data about denosumab rechallenge after AFF, while there are recent data in the literature supporting it after ONJ development (23).

AFF is a term given to low-energy fractures along the diaphysis of the femur, from distal to lesser trochanter to proximal to the supracondylar flare, occurring in patients undertaking antiresorptive drugs (like denosumab and bisphosphonates) (22). Minimal traumas, like a fall from a standing height, may be sufficient to provoke them. It is estimated that AFF occurs in 3–50 cases per 100,000 person-years in patients undergoing antiresorptive therapy, but its true incidence remains elusive, and it appears to increase for long-term use (more than 5 years) (24). Incidence of AFF in patients receiving denosumab appears to be similar to the one observed for other antiresorptive drugs. A phase III randomized FREEDOM trial evaluated 2,626 women treated with denosumab for osteoporosis over a 10-year time span, finding a very favorable risk–benefit profile and an AFFs’ incidence, which was almost negligible. One should recall that dosage in this setting (60 mg every 6 months) is very different than with GCTB treatment (120 mg/month) (25). Dosing schedules similar to GCTB are instead adopted for metastatic cancer patients: in this setting, reported AFF incidence is extremely low (3–50 cases per 100,000 persons per year), but if denosumab therapy is protracted over several years, the risk may be substantially higher (100 per 100,000 persons per year) (21, 25). Accordingly, a recent systematic review by Takahashi et al. found that incidence rate in patients undertaking denosumab therapy due to oncological reasons (therefore, at 120 mg monthly) is approximately 2% (26). In addition, there are compelling data on the association between AFFs and glucocorticoids (22, 27–31). Other risk factors appear to be Asian ethnicity, obesity, and vitamin D deficiency, which were lacking in the case reported herein, but genetic factors could play a role as well (24, 32). Exaggerated suppression of bone turnover by denosumab is the likely cause of fracture formation (26). AFF can be classified as complete if it extends through both cortices, as in our case, or, alternatively, incomplete, when involving lateral cortex only. From a clinical point of view, it is often bilateral, typically preceded by prodromal symptoms such as dull pain in the groin or thigh area and may be associated with delayed fracture healing (a phenomenon termed “delayed union”). AFFs are also characterized by peculiar radiographic signs, such as periosteal callus formation (referred as “beaking” or “flaring”), transverse or short oblique fracture line, and, usually, lack of comminution (22). Early diagnosis can be made with single-energy X-ray absorptiometry (SXA, different from the better-known dual-energy X-ray absorptiometry, or DXA) scan technology, as it could better detect “beaking” sign and early signs of impending fracture and may be periodically offered to patients requiring long-term antiresorptive therapy (33, 34).

Preventive strategy may consist of drug holidays, as AFF risk substantially decreases after antiresorptive drug cessation; although this approach could be an option in the osteoporotic setting, it may not be a solution for patients with GCTB following disease progression. Upon AFF identification, current clinical practice guidelines generally recommend denosumab discontinuation. Internal fixation with intramedullary nailing is the treatment usually adopted for complete AFF management, along with adequate calcium and vitamin D supplementation. In our case, a complete AFF occurred, so that we adopted the forementioned approach. On the other hand, the best management for incomplete AFF is still unclear. The non-operative treatment consists of partial weight bearing, discontinuation of the bisphosphonates, and prescription of supplements (calcium and vitamin D). However, Koh et al. previously reported that the non-operative treatment fails in nearly half of the cases requiring further surgery (35). By contrast, Wang et al. reported that prophylactic surgery results in 97% rate of healing and very few complications (36). The prophylactic surgery is aimed to alleviate pain and to prevent the secondary displacement and/or complications associated with a complete fracture, including delayed union, non-union and implant failure. For these reasons, prophylactic surgery for an incomplete fracture may be justified in selected patients, such as patients with bilateral disease, persistent pain, and/or a previous fracture on the opposite side (36). In this respect, Jiang et al. performed a cost-effective analysis to investigate the role of contralateral prophylactic femur fracture fixation after a bisphosphonate-associated AFF (37). The model suggested that the procedure is cost effective in patients between 60 and 89 years of age with more than one risk factor, such as Asian ethnicity, prodromal pain, varus proximal femur geometry, femoral bowing, or radiographic changes such as periosteal beaking and a transverse radiolucent line. Bony callus formation and healing will likely be slower, and there is weak evidence supporting the prescription of teriparatide (a PTH-recombinant agent promoting bone formation) to accelerate this process, such that its routine employment is controversial (22, 38, 39). Of note, studies indicate that possible rebound vertebral fractures may occur as a result of denosumab discontinuation. European Society for Medical Oncology (ESMO) clinical practice guidelines recommend extreme caution when considering discontinuation of denosumab in metastatic cancer patients (21).

Currently, there are limited data on denosumab rechallenge at disease progression in advanced GCTB, since in common practice, denosumab is mainly administered for clinical conditions such as osteoporosis and metastatic solid tumors in which it is not a life-saving agent, as it is for advanced GCTB. One may well be hesitant with denosumab rechallenge, and, in addition to that, common sense might suggest that denosumab may slow bone healing. Although this may be partially true, it was reported that this mAb is also associated with increased deposition of woven and mature bone (19). In addition, it represents the only potentially active systemic treatment for GCTB. Our group already reported on a case series where denosumab was safely re-introduced after osteonecrosis of the jaw, achieving good disease control, but we could not find reports on denosumab rechallenge after AFF (23). In the case presented herein, as disease progression was documented 14 months from its discontinuation due to AFF, in consideration of the sustained benefit from denosumab before the complication occurred, and because bone healing was complete, we re-introduced denosumab with a new disease stabilization still enduring after 1 year.

As a serious adverse event requiring treatment interruption, it would be worth understanding whether different dosing schedules may reduce the risk for AFFs, whose risk increases over time. Current clinical practice guidelines for GCTB recommend three 120-mg subcutaneous injections during the first cycle (at days 1, 8, and 15), followed by 120-mg injections once monthly. In the lack of prospective data, for unresectable and especially metastatic GCTB, current guidelines suggest that the treatment needs to be continued until evidence of disease progression or toxicity. The balance between maintaining a prolonged disease control and the risks of adverse events due to a chronic treatment is a major unsolved question that brings the attention towards “drug holidays,” as it was explored for other settings to prevent ONJ or AFF. In GCTB, this would need to be answered prospectively, as denosumab is the only agent with proven antitumor activity in the disease, and it is not known if treatment holidays could induce secondary resistance (20, 40–43). Unfortunately, a multicenter, open-label, phase II randomized trial (the “REDUCE” trial, NCT0360149) by the European Organization for Research and Treatment of Cancer (EORTC) designed to answer this question in GCTB was prematurely terminated due to poor accrual. It is therefore unlikely that the question will formally be answered soon.

**Figure 4:**
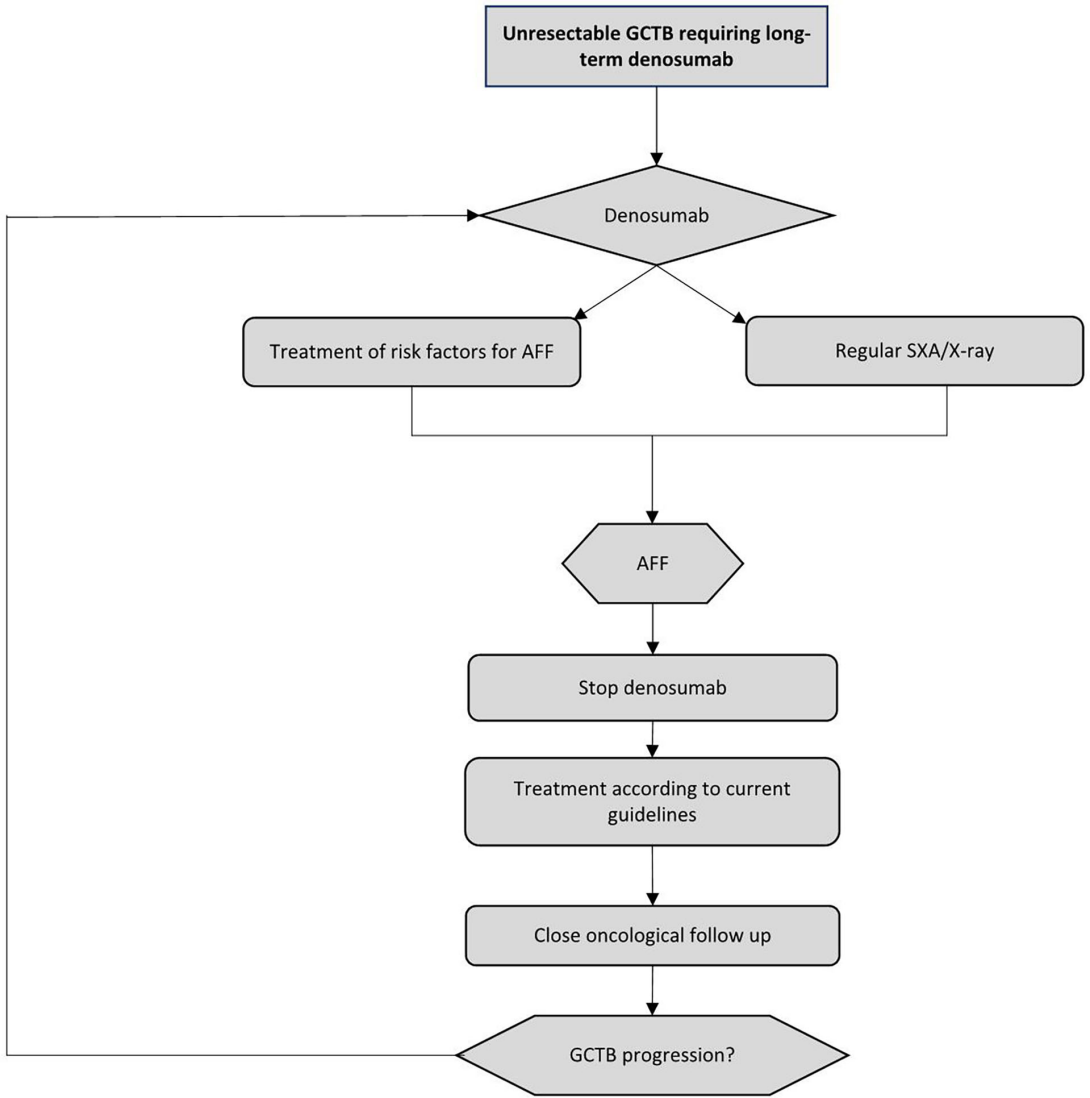
Proposed clinical algorithm for the management of AFF during denosumab treatment in unresectable giant cell tumor of bone (GCTB) patients. AFF, atypical femur fracture; SXA, single-energy X-ray absorptiometry.

In **Figure 4**, we propose a brief clinical algorithm for the management of denosumab-related AFF. Of course, identification of risk factors and their mitigation/elimination would be useful, and on-treatment surveillance for patients at a higher risk is warranted; to this end, if available, SXA could be regularly (once every 6–12 months) offered to patients who have been treated with denosumab for more than 5 years. If diagnosed, a complete AFF should be treated according to current clinical practice guidelines, that is, internal fixation with intramedullary nailing, and denosumab should be discontinued. Prophylactic surgery for an incomplete fracture may be considered in selected patients. If a patient is asymptomatic, this may not be justified. After recovering, which will likely be slower than normal, patients should be carefully monitored for potential disease progression, which variably occurs in almost one half of cases. If there is evidence of GCTB progression, providing the complete resolution of the fracture, we believe that denosumab rechallenge might be a reasonable and safe choice, to be individualized and shared with the patient, balancing the possibility of an AFF recurrence with the need for disease control. Accordingly, strict monitoring for any signs and symptoms indicative of AFF relapse should be undertaken, as the risk may be higher in patients who have already experienced an AFF (25, 44). In case of AFF reoccurrence, the same pathway could be followed, even though no such cases have been reported so far.

In conclusion, AFF can occur in patients on denosumab for long. Our case recalls that it is manageable with denosumab discontinuation and local treatment, even though the healing of the fracture can take longer than expected in patients not treated with this compound. As well known, GCTB tends to progress after a while from treatment stop, but can be safely restarted, achieving new disease control. Thus, an option of denosumab rechallenge even in patients who experienced ONJ or AFF may be clinically attractive. While our case suggests that it is feasible also in AFF, joining efforts for the creation of a global registry for GCTB would provide those real-world data about long-term outcomes and toxicity, which would be definitely needed to allow reliable standard recommendations.

## Data availability statement

The original contributions presented in the study are included in the article/supplementary material. Further inquiries can be directed to the corresponding author.

## Ethics statement

Written informed consent was obtained from the individual(s) for the publication of any potentially identifiable images or data included in this article.

## Author contributions

SS and VN designed the study. AF, CM, CB, SS, and VN collected and analyzed the data. AF, ML, SS, and VN drafted the manuscript. All the authors critically reviewed the manuscript. SS, PC, and ML supervised the final work. All listed authors read and approved the final version of the manuscript.

## Conflict of interest

AF, PC and SS perceived funds for institutional research and grants from Advenchen Laboratories, Amgen Dompé, AROG Pharmaceuticals, Bayer, Blueprint Medicines, Daiichi Sankyo, Deciphera, Eisai, Eli Lilly, Epizyme Inc, Glaxo, Karyopharm Pharmaceuticals, Novartis, Pfizer, PharmaMar.

The remaining authors declare that the research was conducted in the absence of any commercial or financial relationships that could be construed as a potential conflict of interest.

## Publisher’s note

All claims expressed in this article are solely those of the authors and do not necessarily represent those of their affiliated organizations, or those of the publisher, the editors and the reviewers. Any product that may be evaluated in this article, or claim that may be made by its manufacturer, is not guaranteed or endorsed by the publisher.

## References

[1] Strauss SJ Frezza AM Abecassis N Bajpai J Bauer S Biagini R. Bone sarcomas: ESMO–EURACAN–GENTURIS–ERN PaedCan clinical practice guideline for diagnosis, treatment and follow-up☆;. Ann Oncol (2021) 32(12):1520–36. doi: 10.1016/j.annonc.2021.08.1995 34500044

[2] Basu Mallick A Chawla SP. Giant cell tumor of bone: An update. Curr Oncol Rep (2021) 23(5):51. doi: 10.1007/s11912-021-01047-5 33754215

[3] Behjati S Tarpey PS Presneau N Scheipl S Pillay N Van Loo P. Distinct H3F3A and H3F3B driver mutations define chondroblastoma and giant cell tumor of bone. Nat Genet (2013) 45(12):1479–82. doi: 10.1038/ng.2814 PMC3839851 24162739

[4] Werner M. Giant cell tumour of bone: Morphological, biological and histogenetical aspects. Int Orthop (2006) 30(6):484–9. doi: 10.1007/s00264-006-0215-7 PMC3172738 17013643

[5] Noh BJ Park YK. Giant cell tumor of bone: Updated molecular pathogenesis and tumor biology. Hum Pathol (2018) 81:1–8. doi: 10.1016/j.humpath.2018.06.017 29944971

[6] Cowan RW Singh G. Giant cell tumor of bone: A basic science perspective. Bone. (2013) 52(1):238–46. doi: 10.1016/j.bone.2012.10.002 23063845

[7] Roux S Amazit L Meduri G Guiochon-Mantel A Milgrom E Mariette X. RANK (Receptor activator of nuclear factor kappa b) and RANK ligand are expressed in giant cell tumors of bone. Am J Clin Pathol (2002) 117(2):210–6. doi: 10.1309/BPET-F2PE-P2BD-J3P3 11863217

[8] Morgan T Atkins GJ Trivett MK Johnson SA Kansara M Schlicht SL. Molecular profiling of giant cell tumor of bone and the osteoclastic localization of ligand for receptor activator of nuclear factor kappab. Am J Pathol (2005) 167(1):117–28. doi: 10.1016/S0002-9440(10)62959-8 PMC1603441 15972958

[9] Arbeitsgemeinschaft Knochentumoren Becker WT Dohle J Bernd L Braun A Cserhati M. Local recurrence of giant cell tumor of bone after intralesional treatment with and without adjuvant therapy. J Bone Joint Surg Am (2008) 90(5):1060–7. doi: 10.2106/JBJS.D.02771 18451399

[10] Balke M Schremper L Gebert C Ahrens H Streitbuerger A Koehler G. Giant cell tumor of bone: Treatment and outcome of 214 cases. J Cancer Res Clin Oncol (2008) 134(9):969–78. doi: 10.1007/s00432-008-0370-x PMC12160765 18322700

[11] Errani C Ruggieri P Asenzio MAN Toscano A Colangeli S Rimondi E. Giant cell tumor of the extremity: A review of 349 cases from a single institution. Cancer Treat Rev (2010) 36(1):1–7. doi: 10.1016/j.ctrv.2009.09.002 19879054

[12] Algawahmed H Turcotte R Farrokhyar F Ghert M. High-speed burring with and without the use of surgical adjuvants in the intralesional management of giant cell tumor of bone: A systematic review and meta-analysis. Sarcoma. (2010) 2010:586090. doi: 10.1155/2010/586090 20706639 PMC2913811

[13] Gaston CL Grimer RJ Parry M Stacchiotti S Dei Tos AP Gelderblom H. Current status and unanswered questions on the use of denosumab in giant cell tumor of bone. Clin Sarcoma Res (2016) 6(1):15. doi: 10.1186/s13569-016-0056-0 27651889 PMC5022265

[14] Thomas DM. RANKL, denosumab, and giant cell tumor of bone. Curr Opin Oncol (2012) 24(4):397–403. doi: 10.1097/CCO.0b013e328354c129 22581354

[15] Singh AS Chawla NS Chawla SP. Giant-cell tumor of bone: Treatment options and role of denosumab. Biologics. (2015) 9:69–74. doi: 10.2147/BTT.S57359 26203221 PMC4507456

[16] Li H Gao J Gao Y Lin N Zheng M Ye Z. Denosumab in giant cell tumor of bone: Current status and pitfalls. Front Oncol (2020) 10:580605. doi: 10.3389/fonc.2020.580605 33123484 PMC7567019

[17] Chawla S Henshaw R Seeger L Choy E Blay JY Ferrari S. Safety and efficacy of denosumab for adults and skeletally mature adolescents with giant cell tumour of bone: Interim analysis of an open-label, parallel-group, phase 2 study. Lancet Oncol (2013) 14(9):901–8. doi: 10.1016/S1470-2045(13)70277-8 23867211

[18] Rutkowski P Gaston L Borkowska A Stacchiotti S Gelderblom H Baldi GG. Denosumab treatment of inoperable or locally advanced giant cell tumor of bone - multicenter analysis outside clinical trial. Eur J Surg Oncol (2018) 44(9):1384–90. doi: 10.1016/j.ejso.2018.03.020 29650420

[19] Branstetter DG Nelson SD Manivel JC Blay JY Chawla S Thomas DM. Denosumab induces tumor reduction and bone formation in patients with giant-cell tumor of bone. Clin Cancer Res (2012) 18(16):4415–24. doi: 10.1158/1078-0432.CCR-12-0578 22711702

[20] Palmerini E Chawla NS Ferrari S Sudan M Picci P Marchesi E. Denosumab in Advanced/Unresectable giant-cell tumour of bone (GCTB): For how long? Eur J Cancer (2017) 76:118–24. doi: 10.1016/j.ejca.2017.01.028 28324746

[21] Coleman R Hadji P Body JJ Santini D Chow E Terpos E. Bone health in cancer: ESMO clinical practice guidelines. Ann Oncol (2020) 31(12):1650–63. doi: 10.1016/j.annonc.2020.07.019 32801018

[22] Shane E Burr D Abrahamsen B Adler RA Brown TD Cheung AM. Atypical subtrochanteric and diaphyseal femoral fractures: Second report of a task force of the American society for bone and mineral research. J Bone Miner Res (2014) 29(1):1–23. doi: 10.1002/jbmr.1998 23712442

[23] Raimondi A Simeone N Guzzo M Maniezzo M Collini P Morosi C. Rechallenge of denosumab in jaw osteonecrosis of patients with unresectable giant cell tumour of bone: A case series analysis and literature review. ESMO Open (2020) 5(4):e000663. doi: 10.1136/esmoopen-2019-000663 32661185 PMC7359187

[24] Starr J Tay YKD Shane E. Current understanding of epidemiology, pathophysiology, and management of atypical femur fractures. Curr Osteoporos Rep (2018) 16(4):519–29. doi: 10.1007/s11914-018-0464-6 PMC6061199 29951870

[25] Bone HG Wagman RB Brandi ML Brown JP Chapurlat R Cummings SR. 10 years of denosumab treatment in postmenopausal women with osteoporosis: Results from the phase 3 randomised FREEDOM trial and open-label extension. Lancet Diabetes Endocrinol (2017) 5(7):513–23. doi: 10.1016/S2213-8587(17)30138-9 28546097

[26] Takahashi M Ozaki Y Kizawa R Masuda J Sakamaki K Kinowaki K. Atypical femoral fracture in patients with bone metastasis receiving denosumab therapy: A retrospective study and systematic review. BMC Canc (2019) 19(1):980. doi: 10.1186/s12885-019-6236-6 PMC6805596 31640606

[27] Girgis CM Sher D Seibel MJ. Atypical femoral fractures and bisphosphonate use. N Engl J Med (2010) 362(19):1848–9. doi: 10.1056/NEJMc0910389 20463351

[28] Feldstein AC Black D Perrin N Rosales AG Friess D Boardman D. Incidence and demography of femur fractures with and without atypical features. J Bone Miner Res (2012) 27(5):977–86. doi: 10.1002/jbmr.1550 22275107

[29] Meier RPH Perneger TV Stern R Rizzoli R Peter RE. Increasing occurrence of atypical femoral fractures associated with bisphosphonate use. Arch Intern Med (2012) 172(12):930–6. doi: 10.1001/archinternmed.2012.1796 22732749

[30] Thompson RN Phillips JRA McCauley SHJ Elliott JRM Moran CG. Atypical femoral fractures and bisphosphonate treatment: Experience in two Large united kingdom teaching hospitals. J Bone Joint Surg Br (2012) 94(3):385–90. doi: 10.1302/0301-620X.94B3.27999 22371548

[31] Dell RM Adams AL Greene DF Funahashi TT Silverman SL Eisemon EO. Incidence of atypical nontraumatic diaphyseal fractures of the femur. J Bone Miner Res (2012) 27(12):2544–50. doi: 10.1002/jbmr.1719 22836783

[32] Roca-Ayats N Balcells S Garcia-Giralt N Falcó-Mascaró M Martínez-Gil N Abril JF. GGPS1 mutation and atypical femoral fractures with bisphosphonates. New Engl J Med (2017) 376(18):1794–5. doi: 10.1056/NEJMc1612804 28467865

[33] McKenna MJ McKiernan FE McGowan B Silke C Bennett K van der Kamp S. Identifying incomplete atypical femoral fractures with single-energy absorptiometry: Declining prevalence. J Endocr Soc (2017) 1(3):211–20. doi: 10.1210/js.2016-1118 PMC5686782 29264478

[34] Khosla S Cauley JA Compston J Kiel DP Rosen C Saag KG. Addressing the crisis in the treatment of osteoporosis: A path forward. J Bone Miner Res (2017) 32(3):424–30. doi: 10.1002/jbmr.3074 28099754

[35] Koh A Guerado E Giannoudis PV. Atypical femoral fractures related to bisphosphonate treatment: Issues and controversies related to their surgical management. Bone Joint J (2017) 99-B(3):295–302. doi: 10.1302/0301-620X.99B3.BJJ-2016-0276.R2 28249967

[36] Wang K Moaveni A Dowrick A Liew S. Alendronate-associated femoral insufficiency fractures and femoral stress reactions. J Orthop Surg (Hong Kong) (2011) 19(1):89–92. doi: 10.1177/230949901101900121 21519086

[37] Jiang SY Kaufman DJ Chien BY Longoria M Shachter R Bishop JA. Prophylactic fixation can be cost-effective in preventing a contralateral bisphosphonate-associated femur fracture. Clin Orthop Relat Res (2019) 477(3):480–90. doi: 10.1097/CORR.0000000000000545 PMC6382193 30394950

[38] Schneider PS Wall M Brown JP Cheung AM Harvey EJ Morin SN. Atypical femur fractures: A survey of current practices in orthopedic surgery. Osteoporos Int (2017) 28(11):3271–6. doi: 10.1007/s00198-017-4155-4 28770273

[39] van de Laarschot DM McKenna MJ Abrahamsen B Langdahl B Cohen-Solal M Guañabens N. Medical management of patients after atypical femur fractures: A systematic review and recommendations from the European calcified tissue society. J Clin Endocrinol Metab (2020) 105(5):1682–99. doi: 10.1210/clinem/dgz295 PMC7121199 31867670

[40] Anagnostis P Paschou SA Mintziori G Ceausu I Depypere H Lambrinoudaki I. Drug holidays from bisphosphonates and denosumab in postmenopausal osteoporosis: EMAS position statement. Maturitas. (2017) 101:23–30. doi: 10.1016/j.maturitas.2017.04.008 28539165

[41] Reid IR Billington EO. Drug therapy for osteoporosis in older adults. Lancet (2022) 399(10329):1080–92. doi: 10.1016/S0140-6736(21)02646-5 35279261

[42] van der Heijden L Dijkstra PDS Blay JY Gelderblom H. Giant cell tumour of bone in the denosumab era. Eur J Canc (2017) 77:75–83. doi: 10.1016/j.ejca.2017.02.021 28365529

[43] Ottesen C Schiodt M Gotfredsen K. Efficacy of a high-dose antiresorptive drug holiday to reduce the risk of medication-related osteonecrosis of the jaw (MRONJ): A systematic review. In: Heliyon [Internet], vol. 6. Cell Press (2020). Available at: https://www.cell.com/heliyon/abstract/S2405-8440(20)30640-X. Apr 1 [cited 2022 Jun 24]. 10.1016/j.heliyon.2020.e03795 PMC7191576 32373730

[44] Drampalos E Skarpas G Barbounakis N Michos I. Atypical femoral fractures bilaterally in a patient receiving denosumab. Acta Orthop (2014) 85(1):3–5. doi: 10.3109/17453674.2013.854668 24171686 PMC3940982

